# Comparison of Transmission Measurement Methods of Elastic Waves in Phononic Band Gap Materials

**DOI:** 10.3390/ma14051133

**Published:** 2021-02-28

**Authors:** Maximilian Wormser, Daniel A. Kiefer, Stefan J. Rupitsch, Carolin Körner

**Affiliations:** 1 Materials Science and Technology for Metals, Friedrich-Alexander University Erlangen-Nürnberg (FAU), 91058 Erlangen, Germany; carolin.koerner@fau.de; 2 Joint Institute of Advanced Materials and Processes, Friedrich-Alexander University Erlangen-Nürnberg (FAU), 90762 Fürth, Germany; 3 Sensor Technology, Friedrich-Alexander University Erlangen-Nürnberg (FAU), 91052 Erlangen, Germany; daniel.kiefer@fau.de (D.A.K.); stefan.rupitsch@imtek.uni-freiburg.de (S.J.R.); 4 Laboratory for Electrical Instrumentation and Embedded Systems, Department of Microsystems Engineering, University of Freiburg, 79110 Freiburg, Germany

**Keywords:** metamaterial, cellular material, electron beam melting, additive manufacturing, laser doppler vibrometry, elastic waves, mechanical metamaterial, phononic band gap, wave transmission

## Abstract

Periodic cellular structures can exhibit metamaterial properties, such as phononic band gaps. In order to detect these frequency bands of strong wave attenuation experimentally, several devices for wave excitation and measurement can be applied. In this work, piezoelectric transducers are utilized to excite two additively manufactured three-dimensional cellular structures. For the measurement of the transmission factor, we compare two methods. First, the transmitted waves are measured with the same kind of piezoelectric transducer. Second, a laser Doppler vibrometer is employed to scan the mechanical vibrations of the sample on both the emitting and receiving surfaces. The additional comparison of two different methods of spatial averaging of the vibrometer data, that is, the quadratic mean and arithmetic mean, provides insight into the way the piezoelectric transducers convert the transmitted signal. Experimental results are supported by numerical simulations of the dispersion relation and a simplified transmission simulation.

## 1. Introduction

Acoustic metamaterials with phononic band gaps have attracted attention in the scientific community for years. Initially, only theoretical models described the existence of photonic, and, later on, phononic band gaps [1,2]. With new manufacturing methods came opportunities to realize intricate metamaterials consisting of periodic unit cells that do not rely on Bragg scattering to exhibit phononic band gaps. In particular, additive manufacturing techniques have created the possibility to create new structure designs of unmatched complexity [3,4,5]. Recent review papers speak for the rise in interest in the topic of acoustic metamaterials [6,7,8,9].

In the experimental examination of acoustic metamaterials and wave-related phenomena, many methods of exciting and measuring mechanical waves have been used. Depending on the intended frequency range, some methods are more suitable than others. In the audible frequency range between 20 Hz to 20,000 Hz, for example, electrodynamic shakers [10,11], impact hammers [12,13], or vibrational speakers [14,15] lend themselves for the creation of mechanical waves. For ultrasound experiments, waves can be excited via magnetostrictive [16,17] or piezoelectric transducers [18,19].

However, these methods require the attachment of a device to excite vibrations. In consequence, the metamaterial system is influenced by the mass and stiffness of the attached device. In addition, the complex and confined behavior of these devices need to be considered. Contactless excitation sources are rare in single-phase metamaterials. A high impedance mismatch between the two phases of a phononic crystal enable Bragg resonances and, therefore, band gaps [20]. Furthermore, the combination of speakers and microphones can be used in two-phase systems that combine Bragg scattering phononic crystal properties with elastic metamaterial properties [21].

Measuring elastic waves can be realized—among other possibilities—via contact transducers [22], piezoelectric transducers [16,19,23], or accelerometers [10,14]. Similarly to the excitation devices, the attachment of a measurement device also changes the properties of the metamaterial. In most studies, the measured parameter is an electrical signal over time.

Therefore, laser Doppler vibrometry (LDV) offers a very suitable method to measure transmitted elastic waves in metamaterials, as has been demonstrated in various studies [11,17,18,24]. LDV measurement devices are contactless, which reduces the influence on the metamaterial itself. With the use of reflective coatings, they can be used on almost any material. Furthermore, they offer the possibility to measure spatially resolved vibrations that can be combined to a bigger picture by scanning an array of points on a surface. Hence, the wave transmission can be described in more detail and two-dimensional phenomena can be observed.

Our aim in this work is to compare different measurement techniques for uni-directionally transmitted mechanical waves at hand of two additively manufactured, open cellular lattices. At the beginning, Finite Element (FE) simulations are performed to assess the general properties that should be expected for the investigated band gap materials. The obtained dispersion relations of the unit cells provide the theoretical band gap positions. The frequency transmission simulations of a 1D array of unit cells resembles a simplified version of the experimental setup. The actual transmission measurements always excite the waves with a piezoelectric plate transducer but differ in the utilized reception method. First, we use a second identical piezoelectric plate as the receiving element, as was done in our previous work [19,25,26]. The other two techniques utilize LDV to record the spatially resolved out-of-plane velocity amplitude using two different methods for post-processing of the obtained data.

## 2. Materials and Methods

### 2.1. Phononic Band Gap Material

#### 2.1.1. Sample Manufacturing and Preparation

The samples in this work have been manufactured via selective electron beam melting (SEBM), a metal powder-bed-based process, which has been covered extensively in the literature [27]. The material used for manufacturing the samples in this work is Ti-6Al-4V. The process inherently creates a rough surface due to partially molten particles. However, the samples achieve a density of above 99.5%.

**Figure 1:**
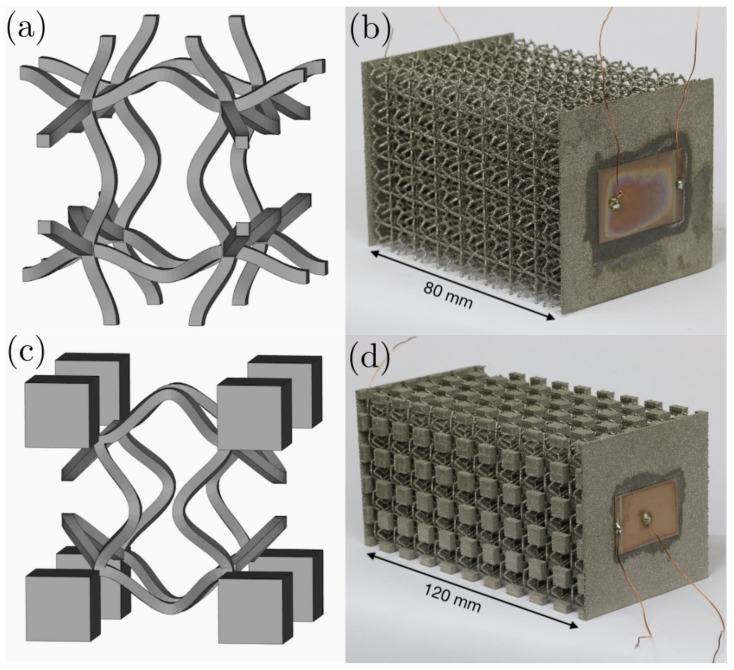
CAD view of unit cell for (**a**) sample 1 and (**c**) sample 2. Photograph of manufactured (**b**) sample 1 with 5.5 × 5.5 × 8 unit cells and a unit cell edge length of 10 mm and (**d**) sample 2 with 5 × 5 × 10 unit cells and a unit cell edge length of 12 mm.

Figure 1 shows the CAD views of the two unit cells and photographs of the samples investigated in this study. The unit cells are composed of 12 intersecting, two-dimensionally shaped struts. For Sample 1, the unit cell is made up of sinusoidal struts that meet in their plateaus. The cellular design was generated by calculating the eigenmode shapes of a simple cubic lattice unit cell with straight struts and periodic conditions [28]. With the eigenmode shape as a template, the cellular design was redrawn in CAD. The full amplitude (from the lowest to highest point) of the struts measures 1 mm.

The unit cell for Sample 2 is derived from Sample 1 with the same sinusoidal strut shapes, however with a larger amplitude of 2 mm, and cubical masses with an edge length of 3 mm that connect to the plateaus of the sinusoidal struts at the corners of the unit cells. The distance between the nearest nodal points is exactly half the unit cell length. The cubical unit cells measure 10×10×10 mm^3^ and 12×12×12 mm^3^ for Samples 1 and 2, respectively.

Both samples feature flat walls on two opposing sides for attaching the piezoelectric transducers. The thin rectangular piezoelectric ceramic transducers (PI Ceramic GmbH, Lederhose, Germany) measure 20×30 mm^2^ with a thickness of 0.2 mm. They were applied to the flat sides of the untreated samples using a thin layer of cyanoacrylate glue. Copper wiring was soldered onto the contacts of the transducers. For this end, the back electrode is extended to one edge on the front side, as seen in the photographs. The samples have been investigated in previous publications [19,25], though exclusively using the piezoelectric measurement setup.

In the same build process that Sample 1 was produced in, a single unit cell with identical parametrical build parameters was produced for measuring the strut thickness via CT scan (μCT 40, Scanco Medical AG, Wangen-Brüttisellen, Switzerland). The strut thickness for Sample 1 was determined to be (0.49±0.12) mm [19]. Since the roughness of the surface does not contribute to the structural stiffness of the strut, the estimation of the mechanically active strut thickness should generally be lower than the measured average thickness for this application [29].

#### 2.1.2. Numerical Evaluation

The dispersion relations were calculated using COMSOL Multiphysics 5.5. An eigenfrequency analysis with Floquet periodicity boundary conditions on a single unit cell was conducted along the path of the irreducible Brillouin zone Γ–X–M–R–Γ [30]. More details on the calculation of the dispersion relations can be found in our previous work [28]. A density of 4420 kg/$m^{-3}$, a Poisson’s ratio of 0.33, and a Young’s modulus of 114 GPa were assumed as material constants for all numerical simulations. Both unit cells feature strut thicknesses that approximate the mechanically active diameter [29] of the manufactured samples. Consequently, Sample 1 has a strut thickness of 0.45 mm that is slightly lower than the average strut thickness measured by CT. Sample 2 has a strut thickness of 0.5 mm.

**Figure 2:**
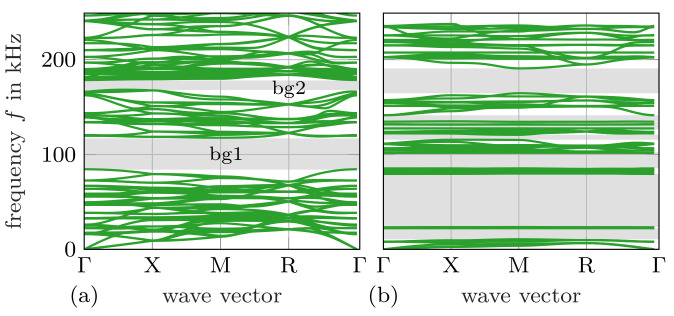
Simulated dispersion curves for (**a**) Sample 1 and (**b**) Sample 2. The resulting band gaps are highlighted in grey.

Figure 2 shows the calculated dispersion relations for Sample 1 and Sample 2. While Sample 1 exhibits two band gaps between 84 and 117 kHz and 168 and 178 kHz, Sample 2 features several band gaps that also cover lower-frequency areas reaching into the audible range, that is, below 20 kHz. The mechanism that enables a pronounced band gap in Sample 1 is a consequence of the way the struts intersect at the nodal points. As opposed to a chiral shape, the way the struts cross in the shown design inhibits rotation, therefore shifting the upper band gap limit to higher frequencies [28].

**Figure 3:**
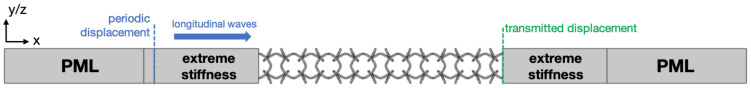
Setup for the transmission simulation. Longitudinal waves are transmitted from left to right. The excitation of the waves is guaranteed to be perfectly longitudinal by domains of extreme stiffness. Perfectly matched layers (PMLs) prevent artifacts from reflections. The transmitted displacement is evaluated at the interface between the cellular array and second extreme stiffness domain. Periodic conditions are applied in y- and z-direction.

The numerical transmission computations were also done using COMSOL Multiphysics 5.5 and are based on frequency domain simulations. A finite, one-dimensional array of seven unit cells (which was found to be the minimum number of unit cells in order to achieve a good depiction of the band gaps) is being excited by strictly longitudinal waves in x-direction, as can be seen in Figure 3. The excitation is created by a harmonic prescribed displacement amplitude of $1\times 10^{-6}$ m. The directions perpendicular to the longitudinal wave propagation (y and z) have periodic boundary conditions applied to them. A material block with extreme stiffness (by a factor of 1000 larger than the Young’s modulus) that is located directly adjacent to the cellular structure ensures that the waves are strictly longitudinal. Both ends of the simulation domain feature perfectly matched layers (PMLs) that absorb waves and prevent reflections. The frequency sweep starts at 1000 Hz and goes up in increments of 100 Hz. Finally, on the other side of the sample where the cellular structure ends, the maximum displacement amplitude is taken as a transmitted signal. The numerical transmission coefficient $T_{\mathrm{num}}$ is calculated at every frequency using the input displacement amplitude $u_{x,\mathrm{in}}$ and the transmitted displacement amplitude in transmission direction $u_{x,\mathrm{trans}}$:

(1) $$T_{\mathrm{num}}=\frac{u_{x,\mathrm{trans}}}{u_{x,\mathrm{in}}}.$$

**Figure 4:**
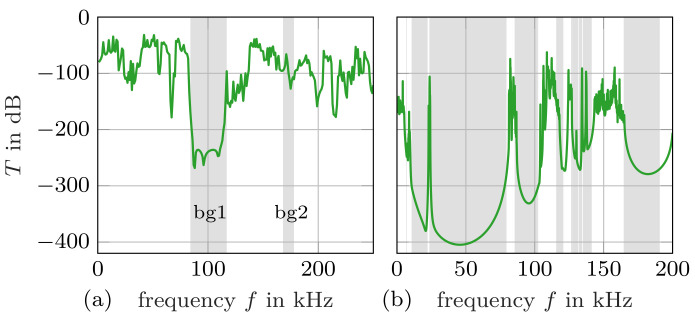
Simulated transmission factor for (**a**) Sample 1 and (**b**) Sample 2. The calculated band gaps are highlighted in grey.

Figure 4 shows the obtained results from the transmission simulations across seven unit cells each. For Sample 1, the first frequency band with low transmission coincides with the predicted first band gap from the dispersion relation. While the second band gap also corresponds to a local minimum in the transmission graph, the attenuation is not as strong. Moreover, there arise several other local minima of similar attenuation levels. The reason for this is that the transmission simulation only covers a single direction, whereas the dispersion relation covers all directions of the irreducible Brillouin zone. Therefore, partial band gaps that occur in a transmission graph of a single direction are omitted in the dispersion relation and the corresponding complete band gaps.

The dispersion relation of Sample 2 exhibits more band gaps and they are also reflected in the transmission graph. Overall, the band gaps predicted by both kinds of simulations align well. Many of the dispersion branches are very flat, that is, their slope is zero. The slope at any point on the dispersion relation reflects the group velocity. Therefore, flat dispersion branches have effectively, or close to no group velocity, which means that there is also very low transmission to be expected. Since, for Sample 2, the dispersion branches are generally flat, the partial band gaps of the transmission simulation and the complete band gaps of the dispersion relation coincide better compared to Sample 1. Consequently, the peaks in the transmission diagram of Sample 2 can also be identified as very narrow breaks in the band gaps given by the dispersion relation. For example, a transmission peak would be expected in the corresponding pass band between 22.3 kHz to 23.5 kHz that is interrupting the second and third band gaps. The transmission diagram shows such a peak just outside that frequency range at 23.7 kHz, which is very close to the expected frequency range.

### 2.2. Measurement Setup and Evaluation

#### 2.2.1. Piezoelectric Transducers

For the transmission measurement with piezoelectric transducers, we connected a frequency generator (HMF 2525, Rohde & Schwarz GmbH & Co. KG, Munich, Germany) to one of the transducers. A sinusoidal signal with a constant amplitude of 10 V was applied in steps of 100 Hz. An oscilloscope (HMO 2024, Rohde & Schwarz GmbH & Co. KG) probed the emitting signal at the actuator and the transmitted signal at the sensor. For each frequency step and both transducers, 64 measurements were taken and averaged. The averaged signal was then smoothed using a Gauss filter (a sigma value of 25 was chosen using the SciPy Python package function scipy.ndimage.gaussian_filter). The difference between the maximum and minimum values of the smoothed signal was determined. Finally, at every frequency, the ratio between the transmitted signal amplitude $A_{\mathrm{trans}}$ and input signal amplitude $A_{\mathrm{in}}$ leads to the experimental transmission coefficient $T_{\exp}=\frac{A_{\mathrm{trans}}}{A_{\mathrm{in}}}$.

#### 2.2.2. Laser Doppler Vibrometry

**Figure 5:**
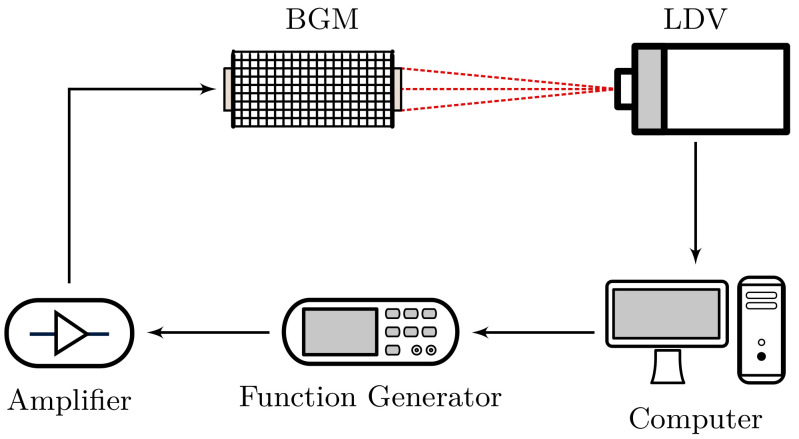
Sketch of the measurement setup with a Laser Doppler Vibrometer (LDV).

The laser Doppler vibrometer (LDV) allowed us to directly attain and analyze mechanical vibrations. The measurement setup is shown in Figure 5. The output of the function generator is amplified, and drives one of the bonded piezoelectric transducers to excite mechanical waves in the band gap material. The other piezoelectric transducer is not used for these measurements. In general, the LDV (PSV-500, Polytec GmbH, Waldbronn, Germany) acquires time-dependent normal surface velocities. The surfaces of both piezoelectric ceramics are scanned by the LDV, resulting in “input” and “transmission” data. Moreover, a personal computer generates the arbitrary excitation signal and also post-processes the data acquired by the LDV.

**Figure 6:**
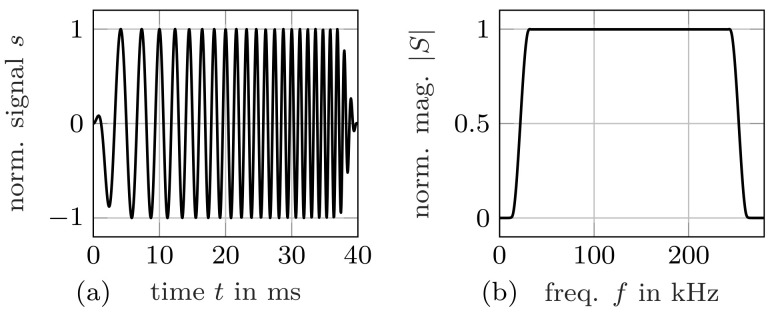
Excitation signal: chirp (**a**) shifted down in frequency for better visualization, (**b**) magnitude spectrum of chirp used for Sample 1.

We utilize a chirp signal for excitation. Figure 6 displays the time-domain signal (shifted down in frequency for visualization purposes) as well as its spectrum. The chirp is designed to cover the frequency band of interest. A long excitation is desirable to increase the time-bandwidth product and, thereby, the signal-to-noise ratio [31]. The maximum duration that does not produce standing waves, that is, multiple reflections in the material, is chosen by a simple longitudinal wave time-of-flight approximation. Moreover, a Tukey (tapered cosine) window with 15% tapering is applied to obtain a smooth spectrum. In our case, these considerations result in a chirp with center frequency $f_{c}=137.5\;\mathrm{kHz}$/110 kHz, bandwidth B=250 kHz/200 kHz, and duration D=40 ms for Samples 1 and 2, respectively.

**Figure 7:**
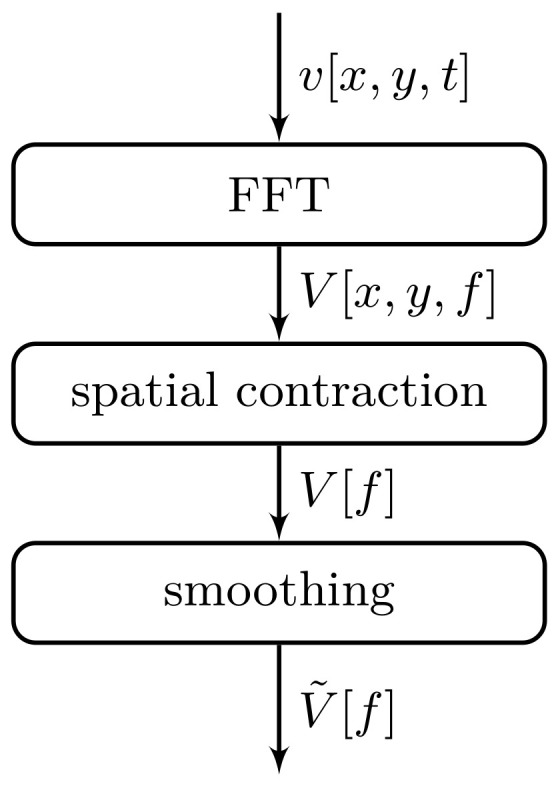
Flow chart showing the steps necessary to obtain the velocity spectra from the LDV data.

The two LDV measurements result in two sets of surface normal velocities, namely, $v_{\mathrm{in}}\left[x,y,t\right]$ and $v_{\mathrm{trans}}\left[x,y,t\right]$. The data sets depend on the two spatial coordinates *x* and *y*, as well as on time *t*. Appropriate signal processing is required to reveal the frequency-dependent transmission factor T[f]. The necessary steps to obtain the two velocity spectra ${\tilde{V}}_{\mathrm{trans}}\left[f\right]$ and ${\tilde{V}}_{\mathrm{in}}\left[f\right]$ are sketched in Figure 7. We start by performing a Fourier transform in time, yielding *N* complex spectra V[x,y,f] for the *N* scan positions ($x_{i}$, $y_{i}$), i=1…N. Next, the spatial dependence needs to be dropped. Different kinds of contractions of the spatial dependence can be performed for this purpose. In this contribution, we analyze and compare two: (a) the quadratic mean given by

(2) $$V\left[f\right]=\sqrt{\frac{1}{N}\sum_{i}V\left[x_{i},y_{i},f\right]V^{*}\left[x_{i},y_{i},f\right]}\;,$$

where $V^{*}$ denotes complex conjugation of *V*, and (b) the arithmetic mean, that is,

(3) $$V\left[f\right]=\frac{1}{N}\sum_{i}V\left[x_{i},y_{i},f\right]\;.$$

These two contractions are different, since the first one ignores the phase of the velocity data, while the second does not. As a last and optional step, smoothing with a Gauss filter is applied, which yields the two smoothed spectra ${\tilde{V}}_{\mathrm{trans}}\left[f\right]$ and ${\tilde{V}}_{\mathrm{in}}\left[f\right]$ that are used to compute the transmission factor

(4) $$\tilde{T}\left[f\right]=\frac{|{\tilde{V}}_{\mathrm{trans}}|}{|{\tilde{V}}_{\mathrm{in}}|}\;.$$

## 3. Results and Discussion

**Figure 8:**
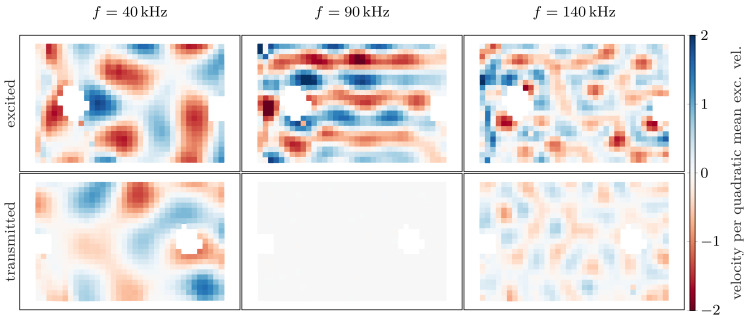
Normal velocity fields scanned by the LDV: The **top row** shows the exciting piezoelectric element at the frequencies marked in Figure 9; the **bottom row** displays the corresponding transmitted fields. The band gap at 90 kHz is clearly visible.

Before analyzing the transmission factors, we present the spatial data obtained by the LDV. The scanned velocity fields are shown for three different frequencies at arbitrary phase in Figure 8. The two spots where the electrodes of the piezoelectric elements had been soldered were omitted in the LDV measurements and appear as blank spots. Note that the resulting fields are not homogeneous, as the piezoelectric element does not excite pure plane waves. For better comparison, the excited and the transmitted velocities have both been normalized to the quadratic mean of the corresponding excited field. One can clearly identify a band gap at 90 kHz in Figure 8, as the transmitted field is approximately two orders of magnitude below the excited one.

**Figure 9:**
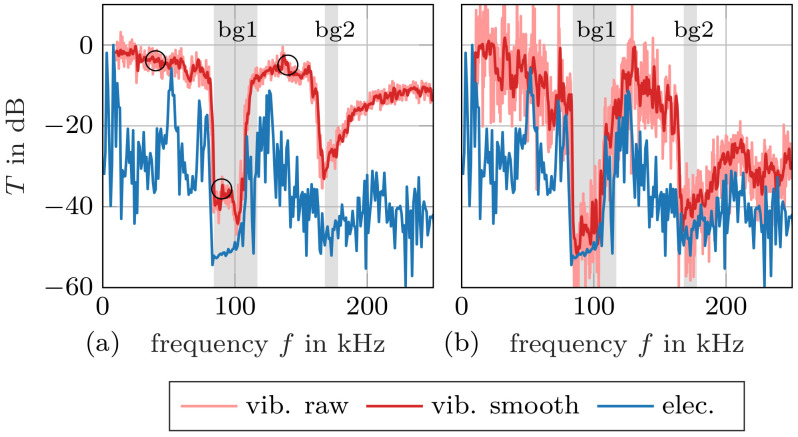
Measured transmission factor for Sample 1: The spatial data of the vibrometer measurements have been contracted with (**a**) the quadratic mean (energy transmission) and (**b**) the arithmetic mean (preserving the phase). The calculated band gaps are highlighted in grey. The circles mark the fields visualized in Figure 8.

**Figure 10:**
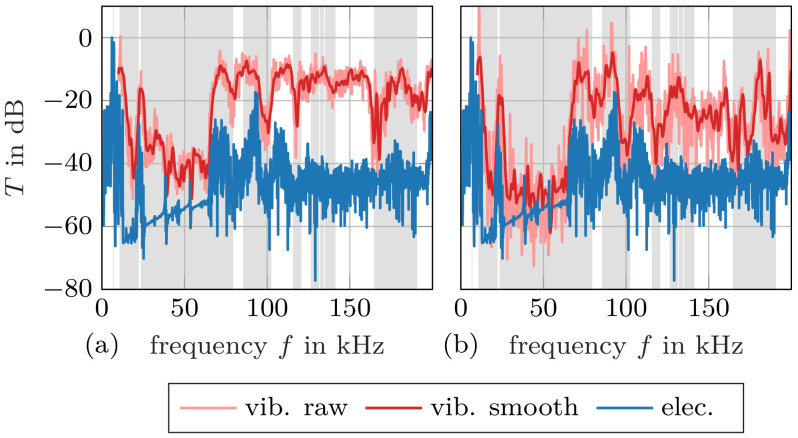
Measured transmission factor for Sample 2: The spatial data of the vibrometer measurements have been contracted with (**a**) the quadratic mean (energy transmission) and (**b**) the arithmetic mean (preserving the phase). The calculated band gaps wider than 5 kHz are highlighted in grey.

After spatial contraction of the LDV data, the frequency-dependent transmission factors can be computed. Transmission factors obtained with the LDV and the piezoelectric transducers are plotted together in Figure 9 and Figure 10 for Samples 1 and 2, respectively. Contraction with the square mean is displayed on the left, while contraction with the arithmetic mean is shown on the right. The calculated band gaps are marked again in the background. Note that the absolute level of the piezoelectric transmission measurements is not known and it is displayed as dB re max. This is of no concern because only the relative level is relevant to identify band gaps. We find a particularly good agreement of calculated and measured band gaps for LDV measurements with quadratic mean contraction (especially Figure 9a). This was expected, since the quadratic mean is proportional to the kinetic energy due to normal velocity of the scanned surface, and the total stored energy determines the transmission factor. It should be noted that part of the mechanical energy may be hidden in the tangential velocity that the LDV is not able to capture.

Inspecting Figure 9b, we find a high similarity between LDV measurements with arithmetic mean contraction and measurements with the piezoelectric elements. The arithmetic mean may lead to cancellations due to regions on the scanned surface vibrating in opposite phase, see Figure 8. This phenomenon is pronounced in the range of high frequencies, where the computed transmission factor according to Figure 9b seems low, though energy is contained in the transmitted signal. As the piezoelectric elements have a single electrode on each side, their response to the distributed mechanical field is a single voltage. This means that the piezoelectric elements perform a spatial contraction of the field that accounts for the local phase of the vibration [32]—similar to our arithmetic mean velocity calculation.

It should also be noted that the predicted band gaps are simulated with a constant strut thickness, whereas in reality, strut thickness can vary significantly, for instance, because of build direction during SEBM. While deviations between numerically and experimentally determined band gaps are small in lower-frequency ranges, the differences become more apparent at higher frequencies. This result is to be expected, since the band gap position scales inversely with the strut thickness. In the case of Sample 1, where there are only two gaps, the deviation is hardly noticeable. However, for Sample 2 with its many band gaps, the fluctuations in strut thickness cause deviations in the band gap positions that are larger than the gaps themselves, therefore creating a mismatch compared to the simulations with uniform struts. Hence, the predicted band gaps do not align as well at higher frequencies, especially for Sample 2.

These results provide insight into the conventional transmission measurements with piezoelectric elements. Our investigations show that such measurements are limited to the low-frequency range, as can be seen in Figure 9a and Figure 10a for both our specimen.

## 4. Conclusions

In conclusion, the comparison of results from numerical calculations, experimental transmission measurements with two piezoelectric transducers, and experimental measurement via LDV showed that all methods can, in principle, identify phononic band gaps. The complete phononic band gaps from the dispersion relation are clearly visible in the numerical transmission diagrams as regions of very low transmission. However, the transmission diagram can exhibit additional partial band gaps that only exist for that particular direction, and this behavior should also be expected for the experimental measurements. The piezoelectric transmission measurements identify regions where band gaps possibly exist. However, the piezoelectric receiving element might inherently exhibit regions of no response, even if mechanical waves are present. This can lead to identification of band gaps that, in reality, do not exist. Exciting the waves with a piezoelectric transducer but measuring it with a LDV delivers spatially resolved transmission data and offers new possibilities for spatial averaging. On the one hand, using the quadratic mean resembles the net energy transmission and gives a frequency-dependent transmission with comparatively low noise. On the other hand, the arithmetic mean preserves the phase of the transmitted waves, similar to single-electrode piezoelectric transducers. Therefore, this kind of averaging resembles the results from the piezoelectric transmission measurement. While transmission measurements via LDV are preferred, for the low-frequency range, it is feasible to resort to the much less expensive setup employing only piezoelectric transducers.

## Data Availability

Data available on request.

## Abbreviations

The following abbreviations are used in this manuscript:

PBG	phononic band gap
LDV	laser Doppler vibrometry
CT	computed tomography
PML	perfectly matched layer
